# Dispersal Capacity and Genetic Structure of *Arapaima gigas* on Different Geographic Scales Using Microsatellite Markers

**DOI:** 10.1371/journal.pone.0054470

**Published:** 2013-01-23

**Authors:** Juliana Araripe, Péricles Sena do Rêgo, Helder Queiroz, Iracilda Sampaio, Horacio Schneider

**Affiliations:** 1 Instituto de Estudos Costeiros, Campus de Bragança, Universidade Federal do Pará, Bragança, Pará, Brazil; 2 Instituto de Desenvolvimento Sustentável Mamirauá, Tefé, Amazonas, Brazil; Ecole Normale Supérieure de Lyon, France

## Abstract

Despite the ecological and economic importance of the *Arapaima gigas* (Cuvier 1817), few data about its dispersal capacity are available. The present study was based on the analysis of microsatellite markers in order to estimate the dispersal capacity of the species on fine, meso, and large geographic scales. For this, 561 specimens obtained from stocks separated by distances of up to 25 km (fine scale), 100 km (meso scale), and 1300–2300 km (large scale) were analyzed. The fine scale analysis indicated a marked genetic similarity between lakes, with low genetic differentiation, and significant differences between only a few pairs of sites. Low to moderate genetic differentiation was observed between pairs of sites on a meso scale (100 km), which could be explained by the distances between sites. By contrast, major genetic differentiation was recorded in the large scale analysis, that is, between stocks separated by distances of over 1300 km, with the analysis indicating that differentiation was not related solely to distance. The genetic structuring analysis indicated the presence of two stocks, one represented by the arapaimas of the Mamirauá Reserve, and the other by those of Santarém and Tucuruí. The dispersal of arapaimas over short distances indicates a process of lateral migration within the várzea floodplains, which may be the principal factor determining the considerable homogeneity observed among the várzea lakes. The populations separated by distances of approximately 100 km were characterized by reduced genetic differentiation, which was associated with the geographic distances between sites. Populations separated by distances of over 1300 km were characterized by a high degree of genetic differentiation, which may be related primarily to historical bottlenecks in population size and the sedentary behavior of the species. Evidence was found of asymmetric gene flow, resulting in increasing genetic variability in the population of the Mamirauá Reserve.

## Introduction

The arapaima (*Arapaima gigas*) is one of the World’s largest freshwater fish scales, with some adults reaching three meters in length and a weight of 200 kg [1]. The arapaima can be found throughout most of the Amazon basin and its tributaries [2], [3], where it is found primarily in lentic habitats, such as lakes and channels of floodplain areas [3], [4]. This important fishery resource has shown signs of overexploitation since the mid nineteenth century, when a significant reduction in numbers was recorded, primarily in the vicinity of the region’s principal urban centers [5]. In recent years, measures have been taken to ensure the conservation of this resource, which include the managed fishery of stocks in sustainable use protected areas, such as the Mamirauá Sustainable Development Reserve (RDSM), Amazonas, Brazil [6], [7]. Participative management has been developed in this conservation unit since 1992, producing satisfactory results, which include an increase in arapaima’s density [8], while the number of fishermen has also increased, along with their gross income [7].

Despite the ecological and economic importance of the arapaima, data on the biological information necessary for the conservation of the species – such as dispersal patterns, reproductive parameters, and taxonomy – are still scarce [4], [9], [10]. Traditionally, the arapaima has been classified as a sedentary fish, with no pronounced long distance movement [4], [11], although in practice, few studies have attempted to quantify the dispersal capacity of this species. Estimates based on molecular data (mitochondrial DNA) [12] suggest intense gene flow among arapaima’s populations located throughout the Amazon basin, which appear to form a single panmictic population on the main channel of the Amazon River. However, analyses of hypervariable microsatellite markers indicate a slight effect of isolation by distance, with restricted gene flow at distances of over 2500 km [13]. Nevertheless, the capacity of dispersion at short distances remains to be clarified. In contrast local dispersal patterns in floodplains are relatively well documented. The várzea environment is characterized by considerable fluctuations in the level of the water, with mean annual amplitude of 10 m and pulses of flooding that have profound ecological impacts on the local fish communities, including arapaima’s populations [3], [14]. The low water levels during the dry season restrict the local fish populations to a small number of habitats, whereas during high water, the flooded area expands, providing access to more abundant feeding resources and refuges. Previous analysis [4] found that the lateral migration of the arapaimas within the várzeas of the Mamirauá Reserve is intimately related to the hydrological dynamics of this environment and the reproductive cycle of the fishes themselves. During the annual period of flooding, the arapaimas leave the lakes and channel systems to which they were restricted at low water and occupy the newly-formed flooded forest habitats. The animals remain in the flooded forest throughout the high water period, where they have access to abundant feeding resources and protection, and return to the lakes as the level of the water declines, completing the annual cycle [4], [10]. During this process of lateral migration, the juvenile fishes migrate to the flooded forest together with the adult male (from April to June), who cares for them over a period of about three months [4]. Once the water level begins to decrease, the adult and juveniles separate and move back into the low-lying habitats. While the dynamics of this lateral migration process are relatively well understood, the patterns of dispersal, vagility, and mechanisms that determine the return of the fishes to the lakes, remain poorly understood.

More detailed data on the dispersal capacity of the arapaima on different spatial scales and dispersal patterns, will be essential to a further understanding of the biology of *A. gigas*, and in particular to the development of effective conservation and management strategies for the species. The purpose of the present study is to complement previous studies [12], [13] using similar highly variable markers but increasing, both sample sizes and collecting sites at distinct spatial scales, in order to provide insights for definition of viable conservation strategies.

## Materials and Methods

The analysis of the dispersal capacity of the arapaima and the structuring of its populations were analyzed on three distinct geographic scales within the natural geographic range of the species. Samples were collected with government approval (process number 02001.007554/2005-76 IBAMA/MMA). Details about the locality and number of samples are showed in Table 1.

**Table 1:** Samples number used in the present study and geographic scales within the natural geographic range of the species.

Locality	Fine (∼25 km)	Meso (∼100 km)	Large (1300–2300 km)
Jarauá I	223	223	223
Jarauá II	−	−	91
Maraã	−	149	149
Santarém	−	−	60
Tucuruí	−	−	38
*Total*	223	372	561

**Figure 1:**
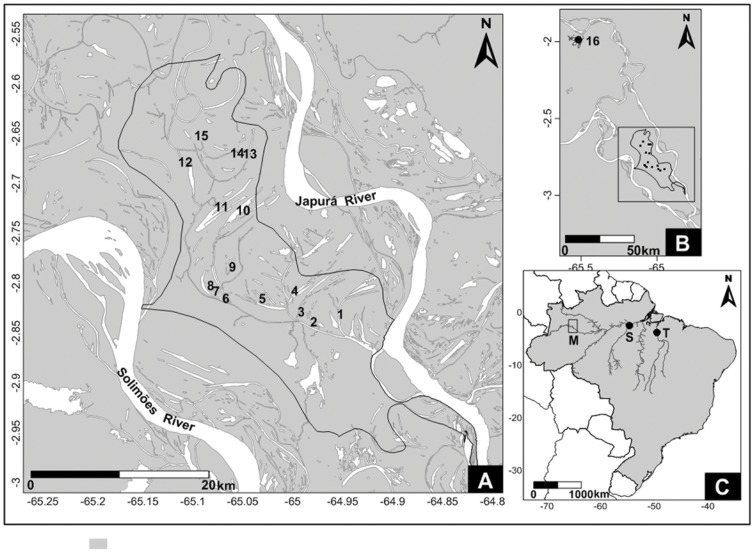
Map showing the localities in three geographic scales. **A**: The locations of the fine scale analysis showing the Jarauá lakes in the Mamirauá Reserve (points 1 to 15). **B**: locations of meso scale analysis showing the Jarauá lakes and Lago Preto Complex in Maraã (point 16). **C**: locations of large-scale analysis showing the Mamirauá Reserve (M), Santarém (S) and Tucuruí (T). The lakes analyzed are: 1- Ressaca do Itú; 2- Ressaca do Curuçá; 3- Ressaca do Panema; 4- Lago do Apuí; 5- Lago Maciel Comprido; 6- Lago Samaumeirinha; 7- Lago Samaumeirinha do Jaraqui; 8- Lago Matá-Matá; 9- Lago Panelão; 10- Lago Jaraqui; 11- Lago Samaúma; 12- Cabeceira do Lago Baixo; 13- Lago Poção; 14- Cano do Cedrinho; 15- Lago Cedrinho; 16- Complexo Lago Preto (Maraã).

Fine scale dispersal patterns were evaluated from the analysis of arapaimas captured in lakes separated by distances of no more than 25 km. For this analysis, specimens of 223 arapaimas were obtained from 15 lakes located within the Jarauá sector of the Mamirauá Sustainable Development Reserve in Amazonas, Brazil (Figure 1A). The meso scale analysis was based on the evaluation of arapaima’s stocks from sites separated by distances of approximately 100 km. This analysis involved the 223 specimens from the Jarauá sector (used in the fine scale analysis) and 149 individuals collected from the Lago Preto lake complex in Maraã, also within the area of the Mamirauá Reserve (Figure 1B). The large-scale analysis compared arapaima’s populations from locations separated by distances of approximately 1300 km and 2300 km. The analysis involved 463 specimens from the Mamirauá Reserve, 60 individuals from Santarem (Pará State) and 38 specimens from Tucurui (Pará State). The 463 specimens from Mamirauá included 223 specimens from the Jarauá sector (used in the fine scale analysis and named Jarauá I), an additional sample of 91 individuals from the same Jarauá sector (named Jarauá II) and 149 specimens from the Lago Preto lake complex in Maraã. The geographic distances between the different sites are shown in Tables 2 and 3.

**Table 2:** Genetic and geographic distances between the lakes analyzed at fine and meso scales.

	1	2	3	4	5	6	7	8	9	10	11	12	13	14	15	16
**1**		3.1	4.4	5.7	8.3	8.9	14.0	14.8	13.1	15.9	17.8	24.2	20.5	21.3	25.0	113.7
**2**	−0.017		1.8	4.0	5.8	6.3	11.3	12.2	10.9	14.7	12.4	22.8	20.0	20.7	24.3	112.8
**3**	−0.016	−0.001		2.4	4.0	4.6	9.7	10.5	9.1	13.0	14.7	21.1	18.5	19.1	22.6	111.0
**4**	−0.012	−0.003	0.025		3.1	3.7	8.7	9.4	7.4	10.7	12.4	18.8	16.1	16.7	20.2	108.7
**5**	−0.007	−0.006	−0.028	0.029		0.6	5.7	6.4	5.2	10.2	12.4	17.7	16.2	16.6	19.6	107.8
**6**	−0.010	−0.002	−0.001	0.010	0.003		5.2	5.9	4.8	10.1	11.2	17.5	16.2	16.5	19.4	107.5
**7**	−0.016	−0.001	−0.006	−0.002	−0.005	−0.003		0.9	3.2	9.5	9.4	14.8	15.8	15.6	17.4	104.3
**8**	−0.013	0.001	0.015	0.001	0.019	0.000	−0.014		3.2	9.1	8.9	14.0	15.3	15.1	16.7	103.4
**9**	−0.026	−0.014	−0.011	−0.007	−0.002	−0.015	−0.030	−0.017		6.4	6.8	12.8	12.7	12.7	15.0	102.8
**10**	0.006	−0.010	0.030*	0.009	0.039	0.017	0.049*	0.029	0.024		2.4	8.4	6.3	6.4	9.5	98.0
**11**	−0.014	−0.014	−0.021	0.005	−0.007	−0.007	0.005	0.011	−0.008	−0.013		6.4	6.7	6.3	8.3	96.4
**12**	−0.012	−0.024	−0.005	−0.010	−0.016	−0.022	−0.027	−0.012	−0.037	0.008	−0.016		7.2	5.9	3.5	90.1
**13**	−0.001	−0.017	0.013	0.027	0.030	0.020	0.032	0.030	0.010	−0.022	−0.006	0.013		1.4	5.8	93.2
**14**	0.019	0.000	0.038*	0.040	0.028	0.029	0.043	0.017	0.012	0.009	0.024	0.004	0.020		4.4	92.4
**15**	0.004	0.015	0.023*	0.029 *	0.015	0.027*	0.006	0.008	−0.004	0.084*	0.040*	0.008	0.058*	0.024		88.5
**16**	0.023*	0.064*	0.070*	0.033*	0.091*	0.050*	0.021*	0.041*	0.020*	0.114*	0.081*	0.045*	0.089*	0.136*	0.064*	

1- Ressaca do Itú; 2- Ressaca do Curuçá; 3- Ressaca do Panema; 4- Lago do Apuí; 5- Lago Maciel Comprido; 6- Lago Samaumeirinha; 7- Lago Samaumeirinha do Jaraqui; 8- Lago Matá-Matá; 9- Lago Panelão; 10- Lago Jaraqui; 11- Lago Samaúma; 12- Cabeceira do Lago Baixo; 13- Lago Poção; 14- Cano do Cedrinho; 15- Lago Cedrinho; 16- Complexo Lago Preto (Maraã).

The pairwise Fst values are shown below the diagonal, and the distances between sites (in km) above the diagonal.

Asterisk (*) indicates as significant value at the 0.05 level after sequential Bonferroni adjustment for multiple comparisons.

**Table 3:** Genetic and geographic distances between sites analyzed within the geographic range of the species (large scale.).

		Fst/geographic distance (in km)			
Population	Sample Size	Jarauá	Maraã	Santarém	Tucuruí
Jarauá	314	−	105	1300	2150
Maraã	149	0.056*	−	1450	2300
Santarém	60	0.146*	0.138*	−	850
Tucuruí	38	0.200*	0.207*	0.068*	−

The pairwise Fst values are shown below the diagonal, and the distances between sites (in km) above the diagonal.

Asterisk (*) indicates as significant value at the 0.05 level.

Samples of muscle tissue were obtained directly from local fishermen, identified, and stored in ethanol until processing in the laboratory. The fishing is intended for marketing and obeys the legislation, which limits the capture to a minimum size of 1.5 m The genetic material collected from the 561 specimens was isolated using the standard phenol-chloroform and proteinase K protocol [15]. Seven informative microsatellite regions (AgCTm4, AgCTm7, AgCAm2, AgCAm15, AgCAm16, AgCAm20, and AgCAm26) were isolated by PCR, using the primers and amplification conditions described previously [16]. The alleles were identified in the Fragment Profiler 1.2 program (Amersham Biosciences) and injected into a MegaBACE automatic sequences (Amersham Biosciences) following the mixture of the amplified product with the ET-ROX 550 standard molecular ladder.

Possible errors in the identification of the alleles were verified using the Microchecker program [17]. Genetic differentiation between the populations was assessed by pair-wise Fst analysis, as well as Φst and AMOVA, run in Arlequin 3.11 [18]. The frequency of private alleles was verified using Genepop 1.2 [19]. The genetic diversity and allele number for each locus were estimated in Genepop [19] while allele richness was calculated in Fstat 2.9.3.2 software [20]. Observed and expected heterozygosis was obtained in Arlequin 3.11 [18]. The meso and large scales analyses were used to estimate the *a posteriori* probability of a given number of stocks based on a Bayesian approach, run in the Structure 2.3.3 program [21,21], which does not require the prior definition of a structure to be tested. The analyses had a burn-in of 50,000, followed by one million replicates of the Monte Carlo Markov Chain (MCMC), the ancestral model with population admixture, and the correlated allele frequency (CAF) model. The number of populations was corrected (ΔK) according to the suggestion of literature [22].

The correlation between the genetic and geographic distances among populations at each spatial scale was estimated using the Mantel test, run in Arlequin 3.11 [18]. The minimum straight-line distances between the lakes located in the Mamirauá Reserve were estimated using the ArcGIS program [23]. These values were considered to reflect the effective distance between lakes, given that during the annual flooding, they are all connected by a continuous body of water [14]. Distances between other sample sites were estimated along the main channel of the Solimões/Amazonas and Tocantins rivers.

## Results

A total of 54 alleles were identified for the seven loci analyzed in the 561 arapaima specimens. The number of alleles, allelic richness, and observed and expected heterozygosities are shown in Table 4. No linkage disequilibrium was detected. The fine scale analysis of the alleles (distances of up to 25 km) revealed that the specimens collected from the 15 lakes of the Jarauá sector were highly similar to each other in genetic terms. Only three of the 45 alleles identified in this sector were unique to a given lake, and the genetic differentiation (Fst) between lakes was significant for only a few pairs, with values ranging from 0.023 to 0.084 (Table 2).

**Table 4:** Indices of genetic diversity for microsatellite markers.

Locus	JARAUÁ (N = 314)				MARAA (N = 149)				SANTARÉM (N = 60)				TUCURUÍ (N = 38)			
	Na	Â	Ho	He	Na	Â	Ho	He	Na	Â	Ho	He	Na	Â	Ho	He
AgCTm4	3	2,997	0.308	0.313	3	2,952	0.486	0.425	4	3,785	0.308	0.313	2	2,000	0.500	0.520
AgCTm7	10	7,258	0.752	0.771	8	7,240	0.817*	0.780	6	5,770	0.752	0.771	5	4,996	0.622	0.652
AgCAm2	12	8,535	0.718*	0.769	7	5,173	0.739	0.700	9	8,604	0.718	0.769	3	3,000	0.324	0.482
AgCAm15	8	6,404	0.653	0.654	8	5, 960	0.524	0.505	4	3,636	0.653	0.654	5	4,842	0.763	0.684
AgCAm16	5	4,764	0.548	0.616	5	4,918	0.550*	0.631	5	4,978	0.548	0.616	2	2,000	0.057	0.084
AgCAm20	4	3,261	0.472	0.538	3	2,418	0.527	0.458	4	3,623	0.471*	0.538	3	3,000	0.447*	0.650
AgCAm26	3	2,996	0.500	0.503	5	3,135	0.480*	0.441	2	2,000	0.500	0.503	2	2,000	0.657	0.466

Na (number of alleles) ¸ Â (allelic richness), Ho (observed heterozygosis) and He (expected heterozygosis).

Asterisk * means significant values (p<0.01).

On a meso scale, 11 alleles were privates to the Jarauá sector, and five to Maraã. Genetic differentiation (Fst) was low to moderate for all the pairs of populations analyzed (Table 2). The AMOVA found an index of differentiation between populations (Φ_ST_) of 0.056, with 4.97% variation between the two sites and more than 90% of the variation within sites.

**Figure 2:**
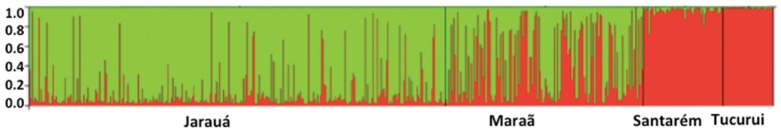
Structure diagram showing the genetic contribution of each stock (ΔK = 2). Each bar represents one individual and the colors green and red represent each stock identified in the present study.

The analysis of large-scale patterns indicated significant genetic differentiation between the localities separated by distances of over 1300 km. The genetic differentiation indices (Fst) varied from 0.138 to 0.207, *p* = 0.00 (Table 3). From the total of 54 alleles identified in the four populations, 17 were exclusive to the Mamirauá Reserve, one was unique to Santarém, and three were found only in Tucuruí. The Mantel test was not significant (p = 0.082), suggesting that genetic (Fst) and geographic distances (in km) are not directly correlated; therefore, the hypothesis of differentiation only by distance between populations can be rejected. The Bayesian analysis also supported the conclusion that the populations furthest apart (over 1300 km) were differentiated from one another, with two distinct stocks being identified within the Amazon basin (ΔK = 2), one in the Mamirauá Reserve, and the other including the populations from Santarém and Tucuruí (Figure 2). The AMOVA indicated that 11.33% of the genetic variation was found among the four localities.

## Discussion

### Fine Scale Analysis

The comparison of the arapaima stocks located in várzea lakes separated by distances of less than 25 km indicated a high degree of genetic admixture. The observed profile indicated that the animals that occupy isolated lakes during the dry season admixture extensively during other periods. Three principal factors related to the biology of the species probably influence this pattern – lateral migration, the reproductive characteristics of the species, and the dispersal pattern of adults and juveniles. The movement of the arapaimas to the recently-flooded forests during the annual period of inundation [4] might result in the admixture of individuals from different lakes. We suggest that they may return randomly to these lakes when water levels fall, instead of returning to their lake of origin. This would explain the intense admixture of alleles observed in the different lakes of the Jarauá sector. This annual movement pattern would renew the population of each lake on a yearly basis and contribute to the homogenization of stocks separated by distances of dozens of kilometers.

Together with this pattern of lateral migration, the reproductive characteristics of the species may also contribute to the marked genetic homogeneity of the populations on a fine scale. The reproductive cycle of the arapaima is intimately related to the hydrological fluctuations of the várzea, and begins at the end of the dry season, when the animals form breeding pairs, build nests, spawn and fertilize the eggs [4], [10], [24]. As the adults in each lake are recycled each year, there is a high chance of forming new breeding pairs each season, which would contribute to an increase in the genetic variability of the population, reinforcing the intense genetic admixture among the lakes observed in the present study.

The third factor that likely contributes to the observed pattern of genetic diversity is the dispersal pattern of juveniles and adults. The males care for the juveniles during approximately three months [10], but they then separate when water levels decline and they return to the lakes when the juveniles – known locally as “bodecos” – are able to feed themselves. Based on the genetic data, these juveniles, which had previously moved no further than one meter from the heads of their fathers [10], begin to disperse within the flooded forest, separating from both the adult male and their siblings during the return to the lakes.

This pattern of dispersal of the juveniles to the lakes and channels adjoining must further reinforce the spread of the different alleles across the várzea habitats. In addition, distinct patterns of dispersal of adult arapaimas in the Mamirauá Reserve have been observed. Specimens have been recaptured or harvested at distances of more than 60 km from the locations at which they were marked and released. On the other hand, some specimens were recaptured in the same (or adjacent) lakes in which they had been released five years previously [24]. Human activities in these lakes may be a primary factor determining this pattern of dispersal, given that these animals may actively avoid returning to impacted environments. In this case, fishery activities in the Mamirauá Reserve may also contribute to the dispersal of the arapaimas, in particular by provoking the emigration of some individuals from the most intensively exploited lakes.

Despite the intense exchange of alleles and an absence of genetic structuring among lakes on a fine scale there is no clear evidence that the arapaimas migrate actively and systematically between different bodies of water. Migration seems to occur at short distances mainly due to lateral migration. However, the pattern of active migration is common in other várzea fish species, such as the silver arowana (*Osteoglossum bicirrhosum*) [25] and the tambaqui (*Colossoma macropomum*), or even the migratory catfish, such as the dourada (*Brachiplatystoma flavicans*), which inhabit the major river systems of the region [26], [27]. According to the genetic data, the adults and juveniles might move slowly and gradually during their lateral migrations, suggesting that the classification of the species as sedentary [28] may not be rejected. The available data on the ecology and behavior of the species, and the dynamics of the várzea floodplains, indicate that the genetic admixture recorded in the present study was related to additional factors other than just migration or dispersal.

### Meso Scale Analysis

A low, but significant degree of genetic differentiation was found between populations on a meso scale distance, that is, approximately 100 km. This also reinforces the relative sedentary nature of the arapaima, given that gene flow between distant sites is relatively low, despite the fact that these environments are linked during the inundation of the várzea floodplains [29]. This conclusion is reinforced by the presence of private alleles in both populations.

The results of the present study indicate that the genetic differentiation observed between the arapaimas of the Jarauá and Maraã sectors may be related to three principal factors – the random dispersal of individuals among lakes, the sedentary behavior of the species, and the effects of distance on these populations. As the flood cycle in this type of environment follows an annual pattern, the dispersal of the fishes to neighboring lakes is limited by this temporal dynamic. Given this, the genetic differentiation observed among lakes on a meso scale has arisen slowly and gradually, reaching significant levels when the distances between lakes approach about 100 km. Each annual flood cycle results in the reconstitution of the population of each lake, including both the adults and the juveniles, as described above. The formation of new breeding pairs, associated with the dispersal of the juveniles results in the dispersal of genes, independently of the migration (*per se*) of the breeding adults.

Despite the sedentary behavior of the arapaimas, then, this model of dispersal appears to result in a gradual, but continuous process of gene flow throughout the várzea floodplains of the Mamirauá Reserve. The dispersal pattern of the alleles does not support the hypothesis that the Jarauá population is virtually isolated, with negligible migration to or from this lacustrine complex, despite the considerable connectivity of these lakes [4]. Despite their reduced dispersal capacity, with movements of approximately 10 km [24], the dispersal of adults and juveniles during the annual flooding cycle appears to permit extensive genetic admixture throughout the várzea floodplains. As this process is gradual, genetic differentiation arises at distances of around 100 km.

### Large-scale Analysis

The analysis of the arapaima populations on the large-scale indicated moderate to high levels of genetic differentiation at distances of over 1300 km. The Mantel test was not significant, indicating that there is not a direct relationship between genetic distance and geographical distance between locations analyzed on a large scale. Probable other factors besides geographical distance contributed to the high differentiation observed. One of them could be the reduction of the population size near to urban center in the region. The present-day pattern of genetic differentiation is supported by the historical records available for the species, which suffered a significant decline in numbers in the populations located in the vicinity of the Amazon basin’s major urban centers [30], [31]. This reduction in population size, associated with the pattern of slow and gradual movements resulted in an accumulation of differences between populations, which led to the significant differentiation observed in the present study between the populations separated by the greatest distances. This scenario is supported by the results of the present study, which identified unique alleles in all the populations, at frequencies of between 0.3% and 28.4% (data not shown). Hrbek et al. [12] reported a similar pattern in analyses of mitochondrial genes and credited it to a severe bottleneck and marked founder effect in these stocks with high levels of genetic differentiation and migration numbers inconsistent with genetic distances between populations.

Analysis of the ATPase and NADH1 genes [12] indicated a lack of genetic differentiation between populations from Mamirauá and Santarém and from Santarém and Marabá which are 1300 Km and 1000 Km far away, respectively. On the other hand, they found higher levels of genetic differentiation between populations, such as Mamirauá and Manaus (approximately 500 km), and Manaus and Santarém (around 600 km), which are much closer than the previous ones. This apparent incongruity was explained by the authors as due to the demographic history of the species, which may have experienced population bottlenecks, in particular in the central part of its geographic range. The findings of the present study indicate that the genetic differentiation of populations separated by distances of more than one thousand kilometers is such that they must be managed separately, and that gene flow is already restricted, even at distances of less than 2500 km as previously proposed by Hrbek [13].

For the implementation of effective conservation measures for the protection of this species from the effects of overfishing, besides the geographic distances other factors must be taken into consideration. One of the most important is the environment in which arapaimas lives, given that some types of behavior presented by the species have major implications for the genetic variability of these populations. Given its ample distribution within the Amazon basin, *A. gigas* can be found in a profuse variety of habitats, which may be responsible for differences in the behavior of this fish. One example is the lateral migration observed in flooded forests, such as those of the várzea floodplains [4], which are less likely to occur in areas such as the Araguaia-Tocantins rivers, where annual fluctuations in water levels are much less pronounced. These variations in environmental factors are likely reflected in considerable differences in the behavior of the species, with obvious implications for the patterns of gene flow among these stocks.

The Bayesian analysis identified two distinct stocks of arapaimas in the different areas surveyed (Figure 2). One stock is formed by the eastern populations (Santarém and Tucuruí), and the other by populations from the Mamirauá Reserve. While Santarém and Tucuruí are located within distinct hydrographic sub basins, these rivers converge in the vicinity of the city of Belém (Pará), which probably facilitates contact between these stocks, given the lack of any major barrier to the dispersal of arapaimas between these areas. However, this result is intriguing and it certainly deserves further studies. Probably new analysis including low Amazon’s and low Tocantins’s populations could clarify this pattern.

The second stock is found in the Mamirauá Reserve, which encompasses a single population characterized by a high degree of genetic variability in the central portion of the Amazon basin. This low-lying region contains ample flooded areas, which provide the arapaimas with refuges, primarily during the low water season. The várzeas floodplains of the Amazon River constitute the largest flooded forest system within the Amazon basin, covering a total area of approximately 200 million hectares [32]. The Mamirauá Reserve represents one of the largest portions of the várzea, with high densities of arapaimas, reaching in some lakes more than 1,000 adults per square kilometer [33], [8]. The conservation of these environments may be reinforcing this refuge effect, given that the Unini and Auati-Paraná Extractivist Reserves, Mamirauá and Amanã Sustainable Development Reserves, together with the Jaú National Park, form a contiguous protected area of almost six thousand hectares. An additional factor that may contribute to the genetic variability recorded in the present study is the fishery management of the arapaima stocks, which has been practiced in the Mamirauá Reserve for more than ten years. The natural difficulties of capturing these fishes in the várzea during the high water period may also contribute to a reduction of the impact of fishing on the natural arapaima populations of this reserve.

The Bayesian analysis indicates an unequal contribution of individuals to the genetic constitution of the different stocks, with the arapaimas from Santarém-Tucuruí (red) contributing more to the Mamirauá population (mostly green) than vice versa (Figure 2). This pattern may be related to two non-exclusive factors – the refuge effect of the Mamirauá Reserve, and the asymmetric gene flow between the two stocks. This pattern of asymmetric gene flow between these two stocks could be supported by the behavioral data. According to Castello [4] “The pirarucu appears to migrate to the flooded forests where males care for the offspring. During the initial and rapid declining of water levels, parental care ceases and the pirarucu migrate back to the river, connecting channels, or lakes where they are fished”. Similar pattern of upriver migrations on the Amazon/Solimões River has been observed in a number of species of migratory siluriform catfish [27]. In the present case, individuals would tend to migrate in the direction of the Mamirauá Reserve more often than towards Santarém or Tucuruí, which lie downriver.

While the arapaima has traditionally been classified as a sedentary fish, no previous studies have analyzed the dispersal capacity of this species over different geographic scales. Together with its dispersal capacity, the effects of the reproductive and behavioral patterns of *A. gigas* on the genetic structure of its populations should be taken into account for the development of effective conservation strategies. In the present study, the indirect analysis of dispersal patterns based on hypervariable genetic markers indicated the occurrence of gene flow between *A. gigas* stocks separated by distances of approximately 100 km in várzea floodplain habitats, with intense admixture on a fine scale (distances of up to 25 km). On the other hand, populations separated by much longer distances, of over 1300 km, were significantly different, indicating a major reduction in gene flow.
